# Supplementary material for: Physiotherapy under pressure: A cross-sectional study on the interplay between perfectionism, moral injury, and burnout
Source: PLoS One. 2025 Feb 3;20(2):e0299173. doi: 10.1371/journal.pone.0299173 (PMC11790081; doi:10.1371/journal.pone.0299173)
Supplement: Attachment — Submitted filename: Considerations.doc [file pone.0299173.s003.doc]

Text

-The use of the Bayeux Tapestry to exemplify perfectionism did not seem within the context for the work in introduction

The introduction is too long and would need to be succinctly rewritten with the main points.

It is important to bring concepts and approaches directly to the profession As this is a research article on a specific population,

Some information is important, such as the means of inviting participants, the approach, the length of time in the profession considered.

The initial part of the discussion presents results.

Some questions could be relevant and be in the discussion: - how does the profession of physiotherapist differ from its equivalents in the United Kingdom? - what are the relevant laws that could support professional physiotherapists? - how do professional councils deal with this, is there any support for physiotherapists?
