# Supplementary material for: Physiotherapy under pressure: A cross-sectional study on the interplay between perfectionism, moral injury, and burnout
Source: PLoS One. 2025 Feb 3;20(2):e0299173. doi: 10.1371/journal.pone.0299173 (PMC11790081; doi:10.1371/journal.pone.0299173)
Supplement: Attachment — Submitted filename: Response to Reviewers.docx [file pone.0299173.s005.docx]

Physiotherapy Under Pressure: A Cross-Sectional Study on The Interplay Between

Perfectionism, Moral Injury, and Burnout

**Response to Reviewers**

| **Reviewer & Editors comments on original document** | **Actioned/Comments** |
| --- | --- |
| **From the word document:** The use of the Bayeux Tapestry to exemplify perfectionism did not seem within the context for the work in introduction | We edited the first paragraph and reduced the use of filter words. We also added a sentence to link the analogy with physiotherapy based on the other reviewer line comments. |
| **From the word document:** The introduction is too long and would need to be succinctly rewritten with the main points.  It is important to bring concepts and approaches directly to the profession As this is a research article on a specific population | We have edited in line with the other reviewers’ comments to embed an increased focus on physiotherapy in the introduction where suggested. We had deliberately focused on the theories of the three main concepts to provide a comprehensive overview in the introduction as it is important for the reader to understand in order to understand the problem in the profession. The detail in the introduction provides a foundation for exploring and making sense of the analysis. |
| **From the word document:** Some information is important, such as the means of inviting participants, the approach, the length of time in the profession considered | Agree information is important and happy review notes it. |
| **From the word document:** The initial part of the discussion presents results. | We strategically reiterate some of the key results to engage the readers with the main findings of the study before we delve into the interpretation. This helps to provide context and align with the central objectives of the study. |
| **From the word document:** Some questions could be relevant and be in the discussion: - how does the profession of physiotherapist differ from its equivalents in the United Kingdom? - what are the relevant laws that could support professional physiotherapists? - how do professional councils deal with this, is there any support for physiotherapists? | While these are important questions, they would deviate from the aim of the current study- to understand the relationship between perfectionism, moral injury, and burnout. If we had only focused on one concept, for example, burnout, then we would have had more scope to unpick the stakeholders involved. We have attempted to partially address this question by adding in ‘Stakeholders like the professional body, the Chartered Society of Physiotherapy (CSP), play a vital role in supporting physiotherapists and offer resources, training, and advocacy on issues such as workplace wellbeing, which includes burnout prevention and management.’ |
| **Abstract:** add comma after shortages | **Actioned:** Suggestion accepted and comma added |
| **Abstract:** Consider rephrasing for clarity and impact; ie " underscores the need for immediate attention to therapists' health and well being". | **Actioned:** Changes made in line with line 25 |
| **Abstract:** Please specify the type of research (ie cross sectional section) to earlier on to inform the reader.  Change aims to "aimed" | **Actioned:** Changes made as suggested |
| **Abstract:** Briefly mention how the online survey was distributed and participants recruited | **Actioned:** Suggestion accepted ‘Participants completed an online survey advertised through social media and distributed in emails. Structure Equation Modelling (SEM) assessed the…’ |
| **Abstract:** Please consider rephrasing for clarity, "Our sample size was calculated to ensure representation of the UK physiotherapy profession, aiming for a 95% CI with a % margin of error. | **Actioned:** Amended for clarity ‘Our sample size calculation represents the UK physiotherapy profession, utilising a 95% confidence interval with a 5% margin of error.’ |
| **Abstract:** Specify what is moderate to high, ie "96% exhibit moderate to high burn out levels" | **Actioned:** Suggestion accepted - ‘96% exhibit moderate to high burnout levels.’ |
| **Abstract:** May be pressed for space in the abstract, but try seeing if you can make this phrase more clear, Ie-- perfectionism and Moral injury, as analyzed through SEM, explain a significant portion (62%) of the variablity in burnout levels. | **Actioned:** Accepted suggestion - ‘SEM revealed perfectionism and moral injury collectively accounted for a substantial 62% of burnout variability, highlighting their sequential impact on burnout manifestation.’ |
| **Abstract:** Urgency could be more effective by briefly specifying the type of interventions that might be considered. (ie Targeted support policies or policy changes) | **Actioned:** Suggestion accepted- ‘With such high levels of burnout, urgent intervention, such as targeted support policies or policy changes, is paramount.’ |
| **Abstract:** Try changing verb use to make more actionable: Ie "Addressing perfectionism and moral injury is pivotal in reducing burn out and improving the well being of physiotherapists". | **Actioned:** Accepted- ‘Addressing perfectionism and moral injury is pivotal to reduce burnout and improve the wellbeing of physiotherapists.’ |
| **Ethics statement:** This seems ambigious and could be problematic from an ethical view point. Please clarify further on how and what they were informed about | **Actioned:** Suggestion taken on board and section edited- ‘Informed consent was provided by selecting the ‘yes’ option when asked for consent to participate in the survey. If they opted yes, they go forward with the survey questions and if they opted no, they would be exited from the survey. ‘ |
| **Ethics statement:** Be more specific on how this was ensured | **Actioned:** Clarity on the process- ‘Anonymity and confidentiality were assured as identifiers, such as names and emails, were not requested. However, at the end of the survey, participants were presented with an option to provide their name and email addresses should they wish to take part in a subsequent qualitative study that builds on the survey results. These identifiers were removed prior to analysis from the master spreadsheet to maintain anonymity.’ |
| **Ethics statement:** Rewrite for clarity "participants could withdraw at anytime without penalty." | **Actioned:** Added in -’The survey explicitly stated the purpose of the study, assured anonymity and confidentiality and emphasised the voluntary nature of participation, with participants' right to withdraw at any time without penalty before analysis made clear.’ |
| **Ethics statement:** This is excellent.. Please comment on whether these were tailored to the potential emotional impacts discussed earlier or if general resources were provided. | **Actioned:** Added the word ‘general’ into the sentence to clarify. It would have been great to have been able to offer more specific resources. Hopefully we will have them in the future. |
| **Line 25:** Please reword for clarity: Ie "necessitates immediate attention to the health and well-being of the therapists." | **Actioned:** Change accepted and made |
| **Line 31:** add comma after professionals | **Actioned:** Change made and accepted |
| **Line 31-33:** Please reword for clarity, ie "a sample size was calculated to ensure a sample of the UK physiotherapy profession." | **Actioned:** Amended to ‘Our sample size calculation represents the UK physiotherapy profession, utilising a 95% confidence interval with a 5% margin of error.’ |
| **Line 34-35:** Please explain further regarding variance in burnout explained by the combination of perfectionism and moral injury | **Actioned:** Edited to ‘UK-based physiotherapists reveals significant burnout levels, with 96% of participants presenting with moderate to high burnout scores.’ |
| **Line 40-42:** Please consider briefly mentioning types of intervention and areas of focus are included. | **Actioned:** ‘such as through amendment or development of policy’ |
| **Line 48-59:** This is a great analogy, but needs to be rephrased to help make a more clear link with the metaphor and the the main themes of your research.  Ie-- "the intricate detail of the Bayeux Tapestry serves as a metaphor for the concept of perfectionism...  Just like the embroiderers of the tapestry, Modern professionals, such as physiotherapists can often strive for similar levels of flawlessness in their work... | **Actioned:** Based on the other reviewer’s feedback and this comment, we reduced some filler words and added in this line ‘Like the embroiderers of the tapestry, modern professionals, such as physiotherapists often strive for similar levels of flawlessness in their work.’ We also edited and moved the original last sentence of this paragraph to the start of the next paragraph to help flow with the edits. |
| **Line 65-67:** Please rewrite for clarity.  For example: This framework shows how elevated self-expectations and maladaptive patterns- stemming from internal pressures can contribute to perfectionist behaviors. | **Partially Actioned**: Enhanced for clarity, however terminology is retained as they were the exact terminology in the framework. |
| **Line 68-70:** Please rephrase for clarity,  For example: On contrast, Hewitt and Flett's model simplifies these into 3 dimensions: | **Actioned:** rephrased to ‘In contrast, Hewitt and Flett’s model3 explains perfectionism as multidimensional and simplifies it into 3 dimensions’ |
| **Line 76-78:** What is the context of this in relationship to physiotherapy?  Also, why is OOP redundant? | **Actioned:** Clarified |
| **Line 81-83:** This is crucial in understanding how maladaptive perfectionism can affect mental and job performance among physiotherapists. | **Actioned:** Agree with the comment. The paragraph following attempts to make this link by connecting maladaptive behaviours of SPP and physiotherapists. |
| **Line 96-102:** Consider rephrasing to streamline concept of SPP and the specific conditions of the UK physios  For example:  This is a form a perfectionism, which is characterized by the pressure to meet multiple expectations. In healthcare, this is problematic when decision making is based on multiple factors.  Practice is shaped by protocols and practioners are caught between the needs of their patients and the healthcare system. | **Actioned:** This is section went through a rephrasing to streamline the concept of SPP and the specific conditions of UK physiotherapists  Edited to include ‘The pressure of multiple expectations partnered with the nature of perfectionism can become problematic in decision making and often cause internal conflict in healthcare. Practice is shaped by protocols and practitioners are caught between the needs of their patients and the healthcare system.’ |
| **Line 104-105:** consider rephrasing "conflict between moral beliefs and the actions they witness and partake in' | **Actioned:** accepted the suggestion. |
| **Line 109-110:** Consider rephrasing... "A violation of a coral moral belief can significantly alter personal identity" | **Actioned:** Suggestion accepted - ‘A violation of moral beliefs can significantly alter personal identity.’ |
| **Line 115:** and/ or compromise their care? Does this in turn effect patient outcomes? | **Actioned:** added potentially compromising the care. Did not include the impact on patient outcomes as it will be a leap. |
| **Line 118:** Or is it "Mirroring" the concept of socially prescibed pefectionism (SPP) | **Actioned:** ‘This mirroring the concept of SPP, where the pursuit of flawless decisions and standards may be hindered by external factors, making the attainment of perfection seemingly unachievable.’ |
| **Line 121-124:** Consider rewording, it is a bit ackward with phrasing and not clear how this is unique to physiotherapists.  Perhaps something like " Cartolovni expands on this by highlighting the conflicts between..." | **Actioned:** edited to ‘Čartolovni et al builds on our understanding of moral injury by highlighting how conflict between a practitioner's duty to provide care, their fundamental professional role, and the witnessing of traumatising events impose an ethical burden on them’ |
| **Line 140:** State the syndrome and what the characteristics are. | **Not actioned:** Authors retained the original text as we feel the structure and wording in this manner prevents repetition further down |
| **Line 150-152:** Consider Rephrasing-  Perhaps "Shirom-Melamed Burnout Measure is also notable for...  which has been validated through confirmatory factor analysis. | **Actioned:** Incorporated the suggestion. |
| **Line 153:** Redundant-- Start with the statement. | **Actioned** |
| **Line 159:** strike out | **Actioned** |
| **Line 160:** in physiotherapist | **Actioned** |
| **Line 168-169:** also to address this issue | **Actioned:** ‘Investigating this aspect is important for advancing our understanding, and allowing for international and interdisciplinary comparisons, and the development of appropriate and specialist interventions.’ |
| **Line 173:** consider changing to correlation? | **Actioned:** Wording changed from relationship to correlation |
| **Line 179:** consider changing to the medical professional and students. | **Partially actioned**: changed to them - to avoid repetition as physiotherapy students was mentioned earlier in the sentence. |
| **Line 183-190:** Simply state what your primary hypothesis is. | **Actioned:** Statement revised to make our thinking / theory more simply stated. |
| **Line 201:** In this study... | **Actioned** |
| **Line 202**: as outlined by... | **Actioned** |
| **Line 202:** Our methodology was... | **Actioned** |
| **Line 211:** add comma after environment.  "work environment, alongside three validated scales... | **Actioned** |
| **Line 214:** consider use full name and abbreviations, as you did it for the first part of the sentence but not for the others (ie MPS-SF and SOP and SPP) OR just use the abbreviations. | **Actioned:** added in full name and abbreviations. The reason we had written it originally like this was because SOP and SPP had been defined in the introduction while the outcome measures had not been introduced yet. |
| **Line 215-216:** rephrase.  consider "due to constraints with the platform..." | **Actioned** |
| **Line 216-217:** the original was adapted... | **Actioned** |
| **Line 218:** (SMBM) | **Actioned** |
| **Line 218:** consider using the full name next to the abbreviation for ease or reading, as well as it keeps the formatting internally consistent, especially with this sentence. | **Actioned:** the reason this was abbreviated was because the terms were not new as they were introduced in the introduction. However, agree re readability suggestion and accepted. |
| **Line 219:** Also required adjustment to accomodate the survey platform | **Actioned**: suggestion accepted |
| **Line 221-222:** rephrase:  Ex-- via social media posts and email campaigns. | **Actioned** |
| **Line 223:** To the MS Forms platform | **Actioned** |
| **Line 225-227:** As stated before, rephrase this to make this more airtight from an ethical standard. Please clarify further on how and what they were informed about | **Actioned.** |
| **Line 230-231:** Rewrite for clarity .  Perhaps simply- "participants could withdraw at anytime without penalty." | **Actioned:** Suggest implemented to provide greater level of clarity |
| L**ine 233-234:** This is excellent.. Please comment on whether these were tailored to the potential emotional impacts discussed earlier or if general resources were provided. | **Actioned:** word ‘general’ added |
| **Lin**e **237-239:** Rephrase for clarity.  Was there a scale used? | **Actioned:** ‘Scores were categorised as low, medium, and high by dividing the highest possible score for each measure into three parts and used third quartile percentages to calculate the three categories’ |
| **Line 247:** (SEM) | **Actioned:** Abbreviation introduced |
| **Line 248-249:** Rephrase--  Like-- a 2 step SEM was applied to evaluate the model fit... | **Actioned:** Rephrased as suggested |
| **Line 249:** add comma | **Actioned:** Accepted suggestion |
| **Line 258-261:** Rephrase. You have 3 sentences that start with the same word.  Comment on the diversity of the settings and duration of employment | **Actioned.** |
| **Line 264-265:** Did you have any exclusion criterion?? | No exclusion criteria. Explained in the limitation’s sections. |
| **Line 267-269:** Rephrase since this is the start of a new header---  For example-- Descriptive statistics including.....  then add where there are located at. | **Actioned.** |
| **Line 269-270:** Slight semantics here, but is this the high or just the maximum score? | **Actioned:** changed to maximum |
| **Line 272-273:** Sounds like there was forced entry with the survey so questions didn't get skipped over, but was there any drop outs or partially filled out responses? | **Actioned:** There was no drop outs and partially filled out data, this clarification is now implemented in the text |
| **Line 290-292:** Rephrase on how this data reinforced your initial hypothesis | **Not Actioned:** This is discussed in the Discussion section |
| **Line 292- 293:** Flush this out further--- relate the findings regarding perfectionism, physiotherapists, moral injury and burn out. | **Not Actioned:** The authors discuss this in the Discussion section in detail, so do not want to repeat information here. |
| **Line 293:** Consider rephrasing to help link the concepts together. | **Actioned** |
| **Line 305:** redundant | **Actioned:** Deleted |
| **Line 306:** what TYPE of perfectionism? | **Actioned:** Clarified as the type of perfectionism is SPP |
| **Line 307:** Again-- the TYPE of perfectonismt combined WITH a moral injury really be the catalyst that drives burnout? | **Actioned:** Clarified as the type of perfectionism is SPP |
| **Line 310:** again be careful with the TYPE of perfectionism (SPP vs OOP vs SOP).  Adaptive (ie normal) perfectionism can be associated with achievement.  Maladaptive perfectionism is negatively associated achievement. | **Actioned** |
| **Line 330:** Please define insomnia further to help with context and relevance | **Actioned** |
| **Line 332-335:** perhaps, but again need to be a little more detailed here. There could be other comorbidities at play depending on the type of insomnia. | **Not actioned:** Brand et al’s work did not clarify the type of insomnia but described it from a holistic view, thus it is difficult to go into greater detail |
| **Line 348:** Again, not to belabor the point, but there are distinct traits between adaptive and malapative perfectionism that could serve as interventions. | **Actioned** |
| **Line 357-360:** May also consider looking at Snyder, C. R., Michael, S. T., & Cheavens, J. S. (1999). Hope as a psychotherapeutic foundation of common factors, placebos, and expectancies. | **Not actioned:** thanks for the suggestion, we will certainly be looking at the next phase of our study where we are looking at interventions. |
| **Line 370-371:** I would argue those who are prone to maladaptive perfectionsim who then experience a moral injury would THEN have a higher risk of becoming burnout. | **Actioned:** rephrased for clarity. |
| **Line 376-379:** Perhaps It could also be from other influences such as their loved ones-- ie Grief-- elements of their life or their loved ones are not what they hoped or planned for. (Fruzzetti 2016) | **Not actioned:** Whilst we see the relevance of grief, adding this can be confusing to the overall message of this study. We will like to keep it focused and not add other factors |
| **Line 403:** What about improving health care delivery to patients? | **Not actioned**: as the strategies are focusing on the healthcare workers and not patients. |
| **Line 403-405:** This is key--- we need to develop a systematic approach to addressing burnout and high pressure environments. This will allow a more healthy and resilient society that can help support individuals well being. from the top to the bottom.  However, there are likely logistical challenges including stakeholder alignment, time and resources needed to complete this. | **We agree with the statement.** We have added a couple of lines on how the professional body can support stakeholder engagement. |
| **Line 408-409:** Should add further the risk of bias with using a convenience sample via social media. Additionally, it can affect the accuracy and generalizability about the findings. Being transparent about the methods and recommendations for future studies help validate your findings.  Addtionally, there is no exclusion criterion, which should be addressed here.  Please explain how the data was cleaned and processed, especially if there were incomplete responses. | **Partially actioned.** The comment on how data was cleaned and processed was N/A as there were no outliers and contradictory questions and all Likert scales were compulsory completions. Therefore, no need to clean and process data. |
| **Line 412-413:** Or-- did this change the sensitivity and specificity, which could impact the accuracy | **Actioned:** included sensitivity and specificity in the sentence. |
| **Line 415-516:** This could have lead to over generalization of prevelance your findings. | **Actioned** |
| **Line 421-322:** Or do a longitudinal study to see if this changes... | **Actioned** |
