# Supplementary material for: Physiotherapy under pressure: A cross-sectional study on the interplay between perfectionism, moral injury, and burnout
Source: PLoS One. 2025 Feb 3;20(2):e0299173. doi: 10.1371/journal.pone.0299173 (PMC11790081; doi:10.1371/journal.pone.0299173)
Supplement: Attachment — Submitted filename: Response to Reviewers 25.05.24.docx [file pone.0299173.s007.docx]

Physiotherapy Under Pressure: A Cross-Sectional Study on The Interplay Between

Perfectionism, Moral Injury, and Burnout

**Response to Reviewers**

| **Reviewer & Editors comments** | **Actioned/Comments** |
| --- | --- |
| **Reviewer #1:** The suggestion is that the conclusion could be more succinct. | Agree. We incorporated reviewer 2’s specific comments which has helped to make this more succinct |
| **Reviewer #2:** Consider Integrating the Bayeux Tapestry into this closing paragraph to help establish a direct comparison between the theme of precision and complexity and the therapist's work.  "This poses a challenge similarly to the embroiders of the Bayeux Tapestry, who meticulously crafted each threat to create a flawless masterpiece. Like the tapestry, physiotherapist must carefully consider numerous factors, meticulously weaving together individual patients needs with the intricate patterns of organizational demands. They are tasked with harmonizing these diverse needs and standards, striving for a tapestry of care that is both comprehensive and precise. However, this intricate balancing act, much like the creation of the tapestry, may introduce elements of unpredictability, potentially leading to discrepancies among the therapist, patient, and the organization, which could ultimately compromise the practioner’s ability to provide optimal care." | Feedback **actioned** with minor amendments to suggestion to enhance flow of entire section. The authors agree with all of the reviewers’ comments in integrating the comparison and feel it has enhanced the paper. |
| **Reviewer #2:** Line 90-92 Consider revising " Strict adherence to SOPs, reminiscent of the painstaking precision required to create the Bayeux Tapestry, can leave healthcare professionals vulnerable to heightened exhaustion and cognitive weariness as they strive for perfection | Feedback **actioned** with minor amendments to suggestion to enhance flow of entire section |
| **Reviewer #2:** Lines 441-442 Consider adding back a subtle connection and reinforce the message: This high prevalence of burnout, akin to a fraying thread in the meticulously woven tapestry of healthcare, has the potential to impact not only the well-being of physiotherapy professionals but also poses risks to patient care, staff dynamics, and the broader society. | Feedback **actioned** with minor amendments to suggestion to enhance flow of entire section |
